# Modeling human migration driven by changing mindset, agglomeration, social ties, and the environment

**DOI:** 10.1371/journal.pone.0264223

**Published:** 2022-02-28

**Authors:** Gonzalo Suarez, Rachata Muneepeerakul

**Affiliations:** Department of Agricultural and Biological Engineering, University of Florida, Gainesville, FL, United States of America; Utah State University, UNITED STATES

## Abstract

Migration is an adaptation strategy to unfavorable conditions and is governed by a complex set of socio-economic and environmental drivers. Here we identified important drivers relatively underrepresented in many migration models—CHanging mindset, Agglomeration, Social ties, and the Environment (CHASE)—and asked: How does the interplay between these drivers influence transient dynamics and long-term outcomes of migration? We addressed this question by developing and analyzing a parsimonious Markov chain model. Our findings suggest that these drivers interact in nonlinear and complex ways. The system exhibits legacy effects, highlighting the importance of including migrants’ changing priorities. The increased characteristic population size of the system counter-intuitively leads to fewer surviving cities, and this effect is mediated by how fast migrants change their mindsets and how strong the social ties are. Strong social ties result in less diverse populations across cities, but this effect is influenced by how many cities remain. To our knowledge, this is the first time that these drivers are incorporated in one coherent, mechanistic, parsimonious model and the effects of their interplay on migration systematically studied. The complex interplay underscores the need to incorporate these drivers into mechanistic migration models and implement such models for real-world cases.

## Introduction

Migration has always been part of human history since the times before we settled for more sedentary lifestyles in farming communities and, later, town centers and urban cities. Humans are driven to move by a diverse set of factors—environmental, social, economic, political, and so on—and these factors influence one another. Human movements exert feedback onto these drivers, e.g., effects on the environment, cultural diversity and social tension, restructuring of labor markets, contributions to and burdens on public infrastructure, etc. These are serious consequences for the well-being of people and societies. As such, there is a need to understand migration dynamics and their relations to these different drivers, and it is no surprise that many theories and models have been developed on the topic—not only for humans (e.g., [1–5]), but also for other social species as well [6–9].

Our initial focus was on modeling migration dynamics in the context of environmental change. It was soon realized, however, that it was not adequate or useful to confine ourselves to “environmentally induced” migration because, as discussed above, the drivers of migration are intertwined [10, 11]. To understand migration *dynamics* and how it is affected by the *interplay* between these intertwined drivers and feedback mechanisms, a migration model with more explicit representation of some of these drivers that captures the dynamics and feedback mechanisms of migration is needed.

Many migration models have been proposed, each with different strengths, disadvantages, and applicability, [1, 12–19] and we have borrowed features of some of these models that fit our goal and made needed modifications. To investigate the interplay between migration dynamics and its drivers in a clear manner, we looked for a minimalist model with a small number of parameters. Parameter-parsimonious population-level migration models come in two broad flavors [1]: *gravity-like*, [20–22] where the migration probability is determined by an equation analogous to the gravitational force between objects, with the mass of an object being replaced by the population of each city and the distance dependence is usually in the form of *d*^−*α*^; and *intervening opportunities*, [23–26] where the migration probability is derived from the relative numbers of opportunities that the potential migrants encounter at candidate destinations. These models share a common idea of using population size as a proxy for factors that drive migration, e.g., job opportunities, productivity, and tendency of humans to agglomerate [27, 28]; this feature helps keep these models parameter-parsimonious, which we considered as strength. However, many of these models have no explicit representation of the environment and therefore no place to incorporate the feedback that populations exert on the environment. Given the enormous pressure that people can put on the environment, such feedback must be part of a model of migration dynamics.

Also lacking in these models is the explicit representation of social ties. Social ties or social networks can strongly influence a potential migrant’s decision on her destination, [3, 11, 29–37] so their effects, too, must be explicitly represented in a model of migration dynamics.

Another assumption in many existing models is that a migrant’s weighing of different drivers remains unchanged throughout a migration episode. This is not always valid. Consider refugees fleeing from a natural disaster or a conflict (e.g., Refs. [38–40]): they may not initially take into account job opportunities or even social ties; they simply want safety and the distance of nearby safe locations may be the only driver during their initial movements. After, say, a few weeks or months, the refugees settle down and feel safer, they may now give more weights to social ties and economic opportunities in the consideration of their next moves. We argue that such changing mindset has long-term consequences on the resulting migration patterns and is thus another important, but often missing, ingredient of a model of migration dynamics.

To address the issues described above, we developed in this paper a minimalist model of migration that includes CHanging mindset, Agglomeration, Social ties, and the Environment (CHASE, for easy reference). In developing this model, we strove to strike the right balance between model simplicity and clearer representation of those drivers of migration. We then conducted numerical experiments with the model’s structure and parameters to investigate how they affect migration outcomes. The scope of this work is to provide insights into the dynamics and interplay between the different aspects of migration included in the model. The analysis enabled us to address the question: How does the *interplay between those drivers and feedback mechanisms* influence transient dynamics and long-term outcomes of migration? Despite its simplicity, the model yielded a rich array of results (some unexpected) that highlight the importance of clearer and better understanding of how these drivers interact to impact migration.

## Methods

### A minimalist migration model: CHASE

**Fig 1:**
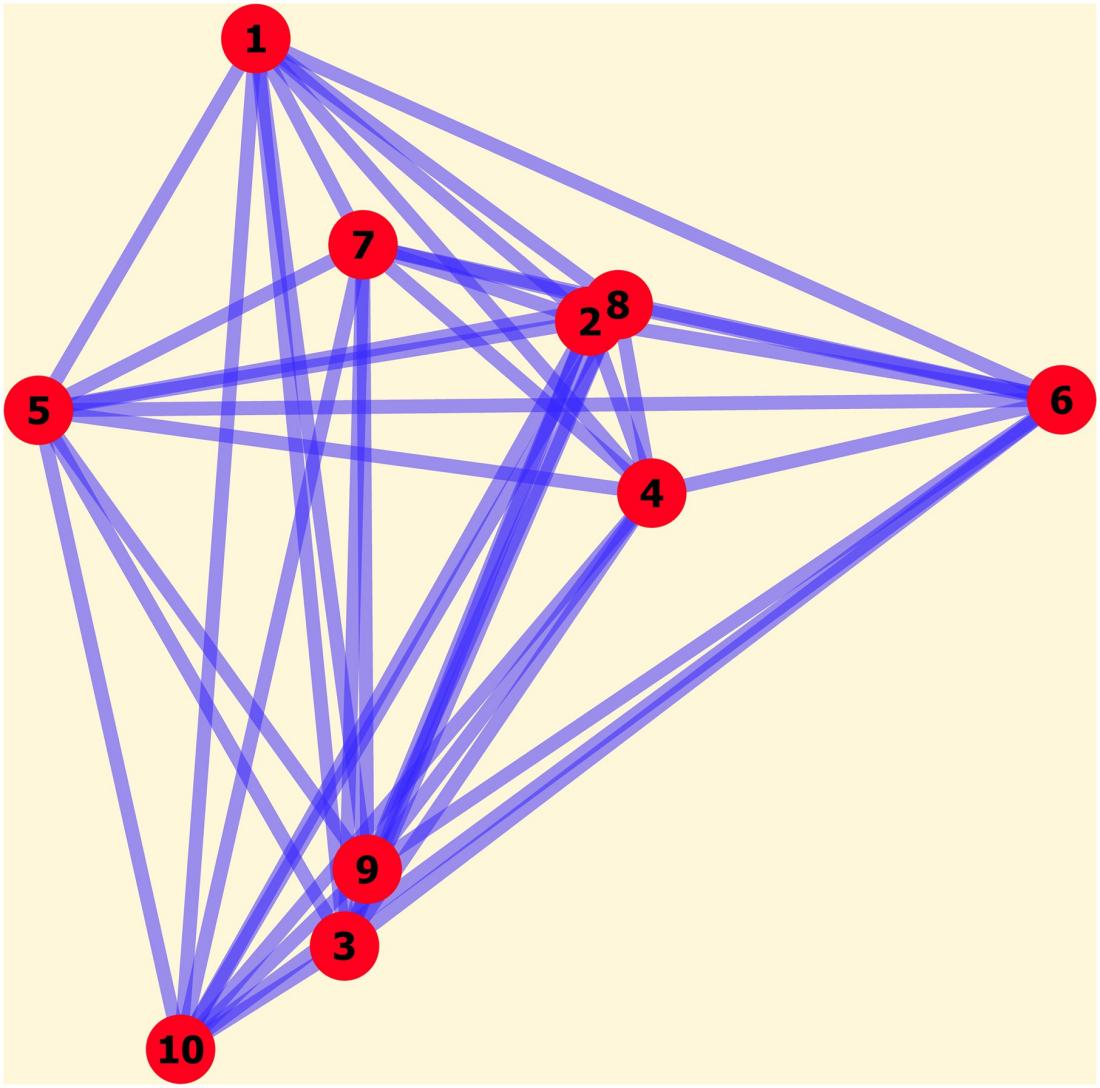
An example of the maps used in the simulations. The cities (red circles) were randomly placed, and people can move between any pair of cities (i.e., a fully connected directed network). This map was used for the population time series shown in Fig 2 and in the S1 Appendix.

In essence, our model is a Markov chain model in which transitional probabilities capture the effects of a few selected drivers of migration process and change over time as individuals move, exerting feedback onto these drivers. The system consists of *L* patches, randomly placed on a two-dimensional plane (see Fig 1 for an example). Each patch represents a “city” with an initial population $n_{j,0}^{\left(j\right)}$ (the term “city” is used for ease of narrative, but one can think of “town” or “village”). A parameter *K*_*j*_ is assigned to city *j*, representing the characteristic population size that the city can support.

Individuals initially in the same town identify themselves as belonging to the same origin/culture/ethnic group. For clarity of result interpretation, it is assumed that the total number of people is constant, i.e., no births and deaths. The probability $w_{ij,t}^{\left(k\right)}$ that an individual of origin *k* moves from patch *j* to *i* in time step *t* is

$$\begin{array}{c} w_{ij,t}^{\left(k\right)}=\{\begin{array}{cc} 1-\delta +\delta {\left(\frac{e}{\beta_{t}}\right)}^{\beta_{t}}{\left(\frac{\sum_{x}n_{j,t}^{\left(x\right)}}{K_{j}}\right)}^{\beta_{t}}\text{exp}\left(-\frac{\sum_{x}n_{j,t}^{\left(x\right)}}{K_{j}}\right)\; & i=j,\\ C\frac{{\left(\sum_{x\neq k}n_{i,t}^{\left(x\right)}+\gamma n_{i,t}^{\left(k\right)}\right)}^{\beta_{t}}}{d_{ij}^{\alpha }}\;\text{exp}\left(-\frac{\sum_{x}n_{i,t}^{\left(x\right)}}{K_{i}}\right) & i\neq j \end{array} \end{array}$$ (1)

where

$n_{j,t}^{\left(x\right)}$ is the number of people of origin *x* in city *j* at time *t*;

*K*_*j*_ represents city *j*’s characteristic population size in the sense that it maximizes the city’s “attractiveness” when *β* = 1—that is, *K*_*j*_ maximizes $\left(\sum_{x}n_{i,t}^{\left(x\right)}/K_{j})\text{exp}(-\sum_{x}n_{i,t}^{\left(x\right)}/K_{j}\right)$—and can be affected by such factors as environmental conditions, food access, armed conflicts, floods, droughts, hurricanes, etc.;

*d*_*ij*_ is the Euclidean distance between cities *i* and *j*;

*δ* is the maximum fraction of emigrating population in one time step—this can be thought of as reflecting the constraints (logistical, financial, political, or otherwise) imposed on the potential migrants: not all who want to migrate can actually do so;

*β*_*t*_ is the exponent that captures the benefit of agglomeration, which can change in time;

*γ* captures the effect of social ties, specifically how much people value others from the same origin;

*α* is the exponent related to the deterring effect of distance (fixed at *α* = 1 as originally proposed by Zipf [20] and used by other models [21]); and

*C* is the normalization constant to ensure that $\sum_{i\neq j}w_{ij,t}^{\left(k\right)}=1-w_{jj,t}^{\left(k\right)}$ (or equivalently $\sum_{i}w_{ij,t}^{\left(k\right)}=1$).

The factor $(e/\beta_{t}{)}^{\beta_{t}}$ ensures that the term following *δ* have the maximum value of 1 and that $1-\delta \leq w_{jj,t}^{\left(k\right)}\leq 1,$ stabilizing the numerical simulations. The $(n_{i,t}^{\left(x\right)}{)}^{\beta_{t}}$ term captures the benefits that arise when people gather together, [28] e.g., economy of scale, extent of public infrastructure, economic prospects, job opportunities, etc.; in addition, one may have a better chance at successfully relocating to a new place if one has connections with people of the same background (think ethnic enclaves)—the $+\gamma n_{i,t}^{\left(k\right)}$ term captures such an effect. On the other hand, the $\text{exp}(-\sum_{x}n_{i,t}^{\left(x\right)}/K_{j})$ term represents the pressure that population put on the city’s support system; the use of the exponential to capture the effects of population on the system’s resources is borrowed from the classic Ricker population model [41]. In principle, *K*_*j*_ may be different for each city *j* and change in time. Indeed, making *K*_*j*_ dynamic and/or a function of social and environmental factors will greatly enrich the model, but it will also complicate result interpretation. Given the exploratory nature of this work, we aimed for clarify by keeping *K*_*j*_ the same for all cities and constant in time, leaving the dynamical and heterogeneous *K*_*j*_ for future work.

### Changing mindset

As discussed earlier, a migrant’s priorities may change during the course of a migration episode. When people escape from a conflict or a natural disaster, they may initially consider only the distance to nearby safer locations and only take into account economic opportunities or social ties in subsequent moves. In order to simulate such changes in priorities, we also explored a version of the model in which the exponent *β*_*t*_ is time-dependant. That is,

$$\begin{array}{cc} \beta_{t} & =1-e^{-t/\tau_{\beta }}. \end{array}$$ (2)

Here, the exponent *β*_*t*_ starts from zero at time *t* = 0 and it asymptotically approaches 1 as time goes by, with *τ*_*β*_ characterizing the speed at which people’s mindsets change. As $\beta_{t}\to 0,(e/\beta_{t}{)}^{\beta_{t}}\to 1$. It follows that at *β*_*t*_ = 0, Eq 1 takes the following simpler form:

$$\begin{array}{c} w_{ij,t}^{\left(k\right)}=\{\begin{array}{cc} 1-\delta +\delta \text{exp}(-\frac{\sum_{x}n_{j,t}^{\left(x\right)}}{K_{j}})\; & i=j,\\ C\frac{1}{d_{ij}^{\alpha }}\;\text{exp}\left(-\frac{\sum_{x}n_{i,t}^{\left(x\right)}}{K_{i}}\right) & i\neq j \end{array} \end{array}$$ (3)

As the migration episode unfolds, people gradually give more weight to, say, economic opportunities and social ties (increasing *β*_*t*_).

### Diversity as a function of social ties

Diversity is an important aspect of a society and it might affect the attractiveness of a region in positive and negative ways [42–45]. To quantify how diverse city *j* is, we use the Simpson’s diversity index [46]:

$$\begin{array}{c} S_{j}=\{\begin{array}{cc} \frac{1}{\sum_{x=1}^{L}{\left(p_{j}^{\left(x\right)}\right)}^{2}} & \text{if}\;\sum_{x=1}^{L}n_{j,T}^{\left(x\right)}>0\\ 0 & \text{otherwise} \end{array} \end{array}$$ (4)

where *T* is the last time step of the simulation and $p_{j}^{\left(x\right)}=n_{j,T}^{\left(x\right)}/\sum_{x}n_{j,T}^{\left(x\right)}$, the fraction of people in city *j* who are originally from city *x*. *S*_*j*_ indicates how well-mixed people from different origins are at the end of the simulations. *S*_*j*_ = 1 means that all the people in city *j* at the end of the simulation was originally from the same city, a completely homogeneous population; *S*_*j*_ = *L* (total number of cities) it means that people from all cities are equally represented in city *j*.

### Parameters used

To clearly investigate the effects of and the interplay among the selected drivers—namely, the migrants’ changing mindsets, human’s tendency to agglomerate, social ties, and pressure people put on the environment—, we varied only the parameters of these drivers, while keeping the rest constant. For each simulation, a new random map with 10 cities was created (an example of which was shown in Fig 1); doing so ensures that the resulting patterns that we observed are robust and not artifacts of any particular configurations of the cities. Unless specified otherwise, *δ* = 0.2, *α* = 1, and $n_{j,0}^{\left(j\right)}=3,000$, the last of which means that each city initially has 3,000 native people and the system’s entire population is 30,000. Each simulation is run for 5,000 time steps. The model was implemented in Matlab, and the code is publicly available in [47].

## Results and discussion

### Legacy effect of the changing mindset

**Fig 2:**
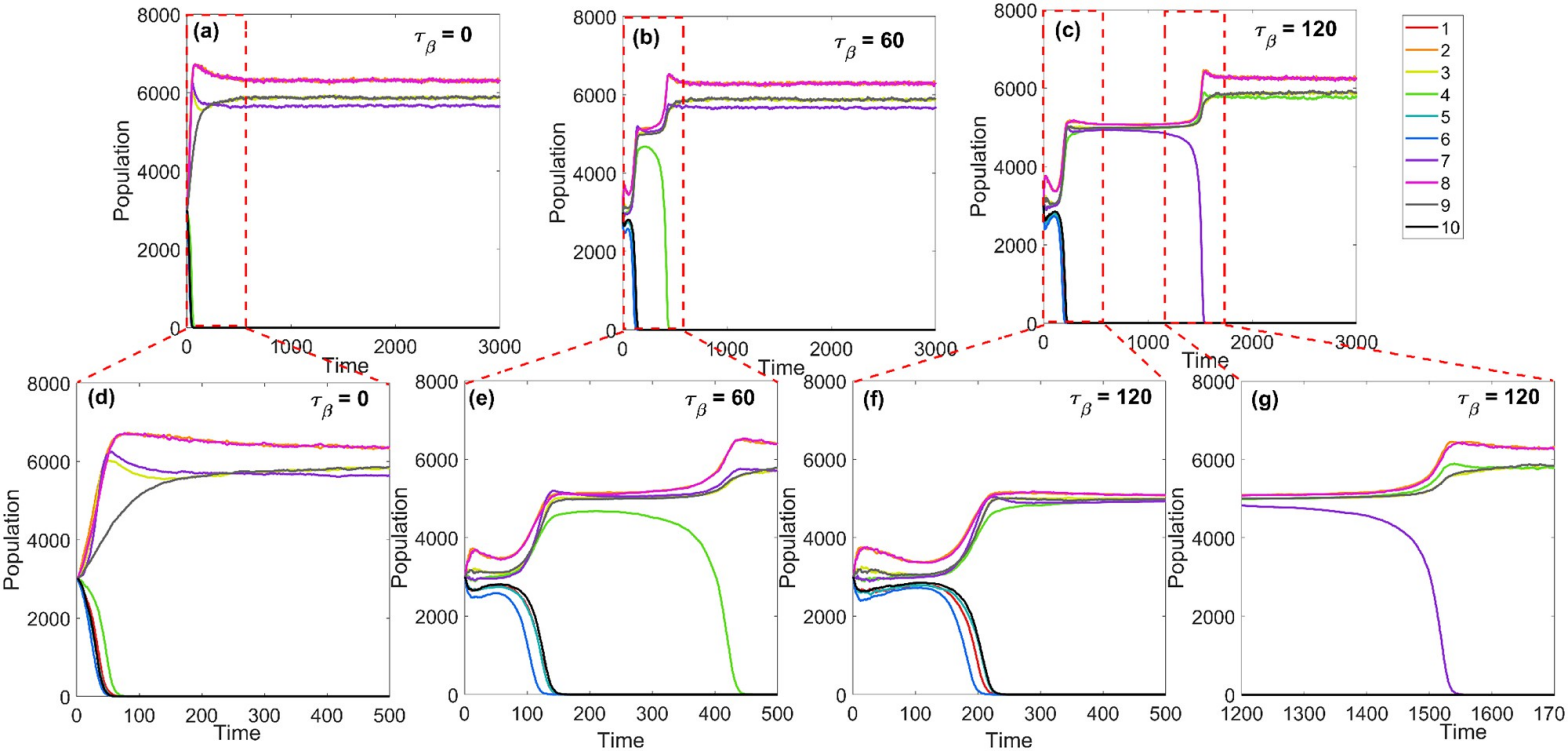
Effects of changing mindset (*τ*_*β*_). Different values *τ*_*β*_ resulted in different transient dynamics of the population time series for each city in the map shown in Fig 1: *τ*_*β*_ = 0Â (*β*_*t*_ = 1) in (a) and (d); *τ*_*β*_ = 60 in (b) and (e); and *τ*_*β*_ = 120 in (c), (f) and (g). The bottom panels show the more dynamic periods of the time series in greater detail. The other parameters are as follows: *γ* = 50, *δ* = 0.2, *α* = 1, *K*_*j*_ = 5000. Ensembles of 100 population time series associated with the parameter sets used in this figure are provided in the S1 Appendix.

As a benchmark, we considered the case in which *τ*_*β*_ = 0, which is equivalent to *β*_*t*_ = 1—people fully take into account job opportunities and social ties from the very beginning (Fig 2a). In this case, the system reaches the equilibrium quickly. When *τ*_*β*_ is greater than 0, there is a change in the migrants’ priorities (*β*_*t*_ increasing from 0 to 1). At *t* ∼ 0, *β*_*t*_ ∼ 0 and migration probabilities follow Eq 3. As time goes by, *β*_*t*_ increases, and the effects of social ties and economic opportunities become more relevant. When *τ*_*β*_ = 60 (Fig 2b), the long-term state of the system is the same as the one in *τ*_*β*_ = 0, but the temporal evolution is different at the beginning. However, for *τ*_*β*_ = 120, not only does the temporal evolution changes, the final state of the system, too, is different (Fig 2c). For example, the population of city 4 (green line in Fig 2) rapidly goes to 0 when *τ*_*β*_ ≤ 60, but it reaches almost 6, 000 when *τ*_*β*_ = 120, and the opposite is true for city 7 (purple line in Fig 2). In other words, the system exhibits “legacy effects,” where transient dynamics (historical pathways) affects long-term outcomes. This highlights the importance of taking migrants’ changing priorities into account, a feature that is often lacking in many existing migration models.

### Higher characteristic population size, fewer surviving cities

**Fig 3:**
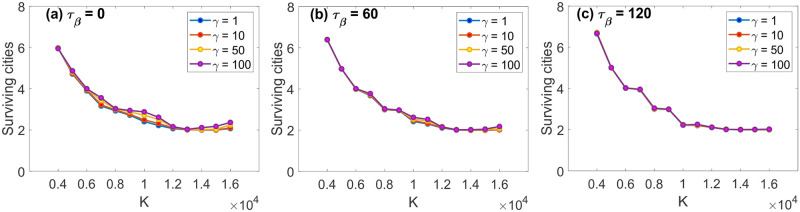
Number of surviving cities as a function of the characteristic population size *K*. The number of surviving cities was averaged over 1,000 runs, using a different random map for each one: (a) *τ*_*β*_ = 0 (i.e. *β*_*t*_ = 1); (b) *τ*_*β*_ = 60; (c) *τ*_*β*_ = 120. A “surviving city” is a city with population greater than 0 at the end of the simulation.

Recall that *K*_*j*_ represents the characteristic population size that city *j* can hold and is assumed to be the same across all cities during the simulation. Our model results show that the number of surviving cities (i.e., cities with population greater than 0) decreases as *K*_*j*_ of each city increases (Fig 3). At first, such a pattern appeared counter-intuitive: Why would fewer cities survive if each can support more people? It turned out that this pattern stems from the “released” benefit of agglomeration (the $(\sum_{x}n_{j,t}^{\left(x\right)}/K_{j}{)}^{\beta_{t}}$ term) as the “cost” of supporting more people (the $\text{exp}(-\sum_{x}n_{j,t}^{\left(x\right)}/K_{j})$ term) is reduced. Put it another way, given sufficient characteristic population size (large enough *K*), people want to agglomerate into bigger groups. This mechanism reminds one of how farming and domestication gave rise to the first villages in human history.

The relationship between the characteristic population size *K* and the number of surviving cities in the system is also influenced by the strength of social ties (*γ*) and how fast migrants change their mindset (*τ*_*β*_). Such interplay is more pronounced for an intermediate range of *K*, around 7,000 to 12,000 when *τ*_*β*_ is small, and becomes weaker as *τ*_*β*_ increases (Fig 3). In this range of *K*, the number of surviving cities is higher for stronger effects of social ties (greater *γ*). In these cases, migrants are more reluctant to leave their native cities for bigger cities, thereby maintaining more cities in the long run. As *τ*_*β*_ is higher, however, the number of surviving cities is less affected by *γ*. A high value of *τ*_*β*_ reflects a scenario where migrants take longer to shift their mindsets to include social ties and economic opportunities in their decision making, thereby reducing the effects of social ties on the resulting number of surviving cities. These results highlight the importance of studying not only the effects of different drivers on migration, but also how they interplay with one another.

**Fig 4:**
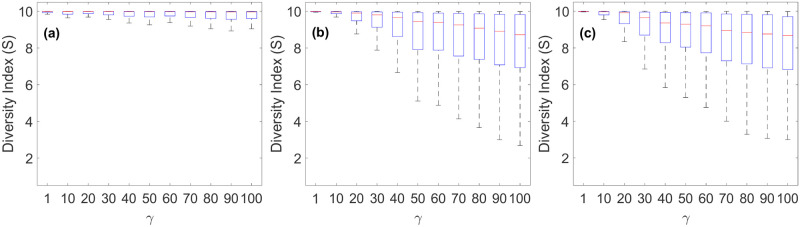
Simpson’s diversity index (*S*) as a function of the strength of the social tie (*γ*). (a) cases with 3 surviving cities, (b) cases with 4 surviving cities and (c) cases with 5 surviving cities. Each box plot represents *S*’s of all surviving cities across different simulations that used the same *γ* and resulted in the same number of surviving cities: central red mark = median; bottom edge = 25^th^ percentile; top edge = 75^th^ percentile; whiskers extend to the most extreme data points not considered outliers [48]. Parameters used in this figure: *K*_*j*_ = {4000, 5000, 6000, …, 12000}, *τ*_*β*_ = 120, *δ* = 0.2, 1, 000 realizations. The results are grouped based on the number of surviving cities because it strongly influences the diversity index (see text).

Finally, we analyzed the diversity of cities and its dependence with social ties using Simpson’s diversity index as defines in Eq 4. When *γ* = 1 there is no special social ties between people with the same background, resulting in well-mixed cities (*S*_*j*_ close to 10; Fig 4). As the effects of social ties become stronger, cities become less diverse, because people aggregate with others from the same origins. The diversity index is strongly influenced by the number of surviving cities; hence the grouping based on the number of surviving cities in Fig 4. To see this, imagine an extreme case in which only one city survives: in this case, regardless of parameters used, *S* will strictly be 10 because everyone—3,000 people from each city—is there. As more cities remain, they are generally less diverse. With more surviving cities, people have more options for destinations and the social ties effects become more pronounced, more nonlinear, and more heterogeneous (Fig 4).

### Limitations and future work

To obtain a clear picture of the effects on migration and the interplay among its drivers, only a few of them were selected and a number of simplifying assumptions were made. Despite its simplicity, the model yielded a rich array of results that highlight the *interplay* of these different drivers. Future work can build on this work by relaxing some of the simplifying assumptions in a number of ways. The characteristic population size *K*_*j*_ can be heterogeneous across cities and have its own dynamics that is governed by other social and environmental variables, describing a more realistic scenario using a bigger number of smaller and larger cities. Changes in the characteristic population size may be gradual or sudden; given the legacy effects found in this work, migration dynamics and long-term outcomes are expected to be different for gradual changes (e.g., prolonged droughts) versus sudden shocks (e.g., armed conflicts or natural disasters) in the characteristic population size. The strength of social ties, could also be heterogeneous across cities; indeed, different cultures value these social connections at varying degrees. Depending on data availability, the model should be modified and implemented for real-world case studies, as both a predictive tool and a theoretical lens through which we look at the empirical patterns. Additionally, the model may be adapted to describe migration of other social species; indeed the comparison of findings from applying the model of the same structure to humans and to other social species will lead to deeper insights towards a general theory of migration. Given the rich interplay suggested in this work, we suggest that these modifications be done in small steps such that the effects of each modification are clear.

## Conclusions

In this study, we set out to address the following question: How does the interplay between the selected drivers—changing mindset, agglomeration, social ties, and the environment—and feedback mechanisms influence transient dynamics and long-term outcomes of migration? We did so by developing and analyzing a parsimonious Markov chain model, dubbed CHASE, that incorporates these drivers. Our model results suggest that these drivers interact in nonlinear and complex ways. Migrants’ changing mindset affects not only the transient period but also long-term outcomes, highlighting the importance of including this feature in migration models. Increased characteristic population size of the system can, perhaps counter-intuitively, lead to fewer surviving cities, and this effect is mediated by how fast migrants change their mindsets and how strong their social ties are. Strong social ties also result in less diverse populations across cities, but this effect is influenced by how many cities remain. The overall results paint a picture of intertwined effects of these drivers on migration dynamics and patterns. To our knowledge, this is the first time that these drivers are incorporated in a mechanistic, parsimonious model and the effects of their interplay on migration is systematically studied. The complex interplay underscores the need for migration models to mechanistically incorporate these drivers. Future research directions include adding more realism into some of the model components: making the characteristic population size of each city dynamical and dependent on other social and environmental variables; making the strength of social ties vary across different cultures; perturbing the system with gradual and sudden changes to compare how the system responds; and modifying and implementing the model for real-world case studies. All in all, we believe the CHASE model constitutes a versatile and novel tool to study migration process in a wide range of scenarios.

## Supporting information

S1 Appendix Ensembles of population time series. Complete time series for 100 independent runs of the model using three different combination of parameters used in Fig 2 and the map shown in Fig 1.
(PDF)
