# Supplementary material for: Modeling human migration driven by changing mindset, agglomeration, social ties, and the environment
Source: PLoS One. 2022 Feb 28;17(2):e0264223. doi: 10.1371/journal.pone.0264223 (PMC8884484; doi:10.1371/journal.pone.0264223)
Supplement: S1 Appendix — Complete time series for 100 independent runs of the model using three different combination of parameters used in Fig 2 and the map shown in Fig 1. (PDF) [file pone.0264223.s001.pdf]

S1 Appendix: Ensembles of population time series

CHASE is a stochastic model, so it is instructive to consider an ensemble of realizations produced by a given parameter set to obtain insights regarding the model's overall behavior and uncertainty and fluctuation around that behavior. Here, we consider ensembles of the time series of populations in the ten cities in the map shown in Fig. 1 with the parameter sets associated with the population time series in Fig. 2 (Figs.S1.1, S1.2, and S1.3).

These additional results revealed that longer timescale of changes in mindset, i.e., larger values of τ_β, lead to more variability and uncertainty in the population dynamics of the cities. This can be seen clearly by comparing the time series in Fig. S1.1 ($\tau_\beta = 0$) to those in Fig. S1.2 ($\tau_\beta = 60$). When the timescale of changes in mindset is large enough (e.g., $\tau_\beta = 120$; Fig. S1.3), the uncertainty can be so much that a city may survive in one realization but collapse in another. In sum, while the conclusions discussed in the main text hold, these ensembles of population time series offer additional insights into the behavior of the CHASE model.

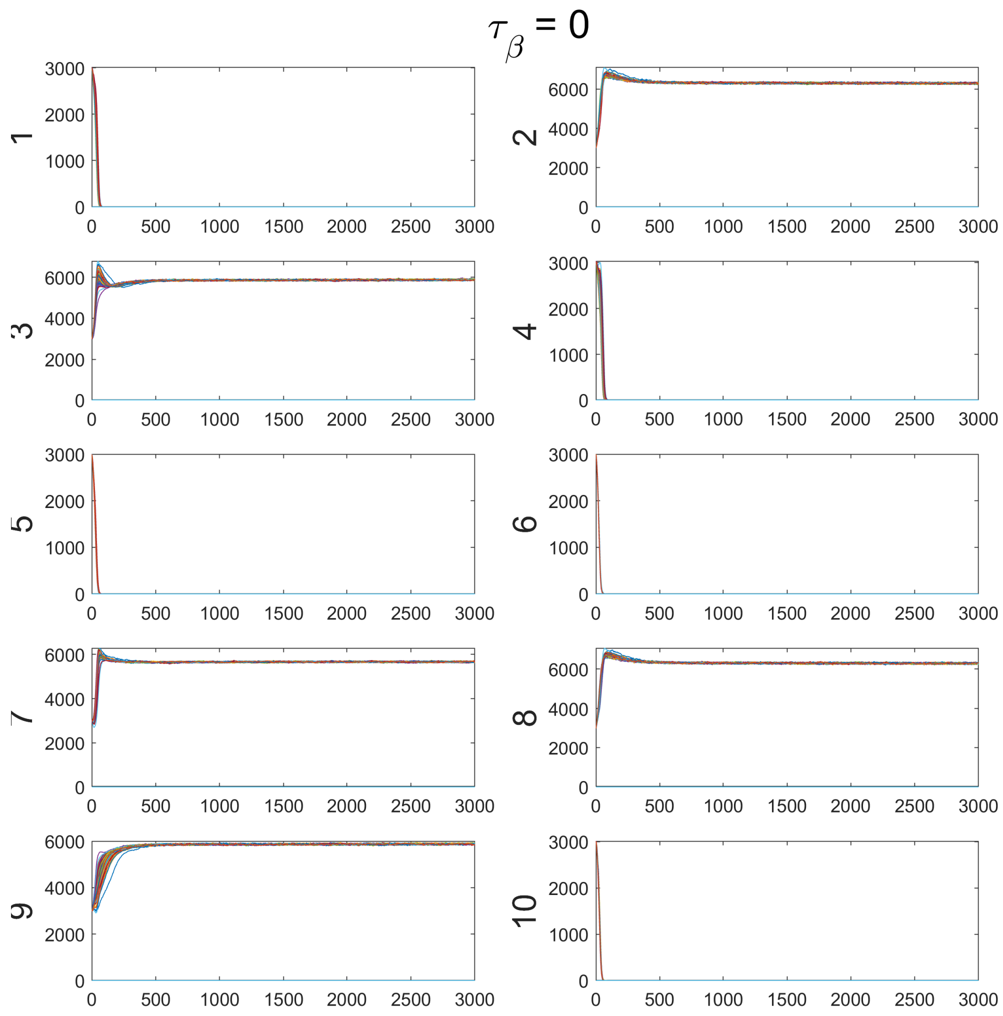

Fig S1.1. 100 realizations of the population time series of each city (1 to 10) for $\tau_\beta = 0, N = 3000,\ K_j = 5000,\ \delta = 0.2,\ \gamma = 50,\ \alpha = 1$, the same parameter set used in Figs. 2(a) and 2(d).

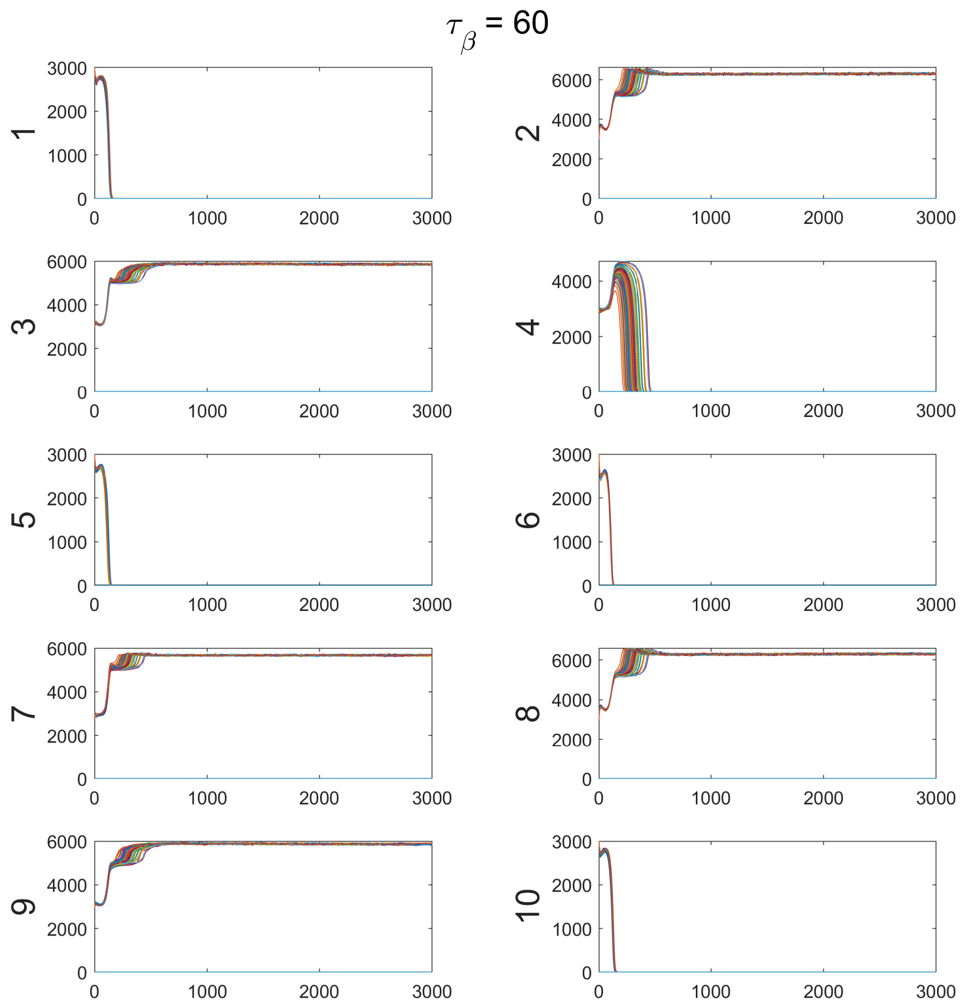

Fig S1.2. 100 realizations of the population time series of each city (1 to 10) for $\tau_\beta = 60, N = 3000,\ K_j = 5000,\ \delta = 0.2,\ \gamma = 50,\ \alpha = 1$, the same parameter set used in Figs. 2(b) and 2(e).

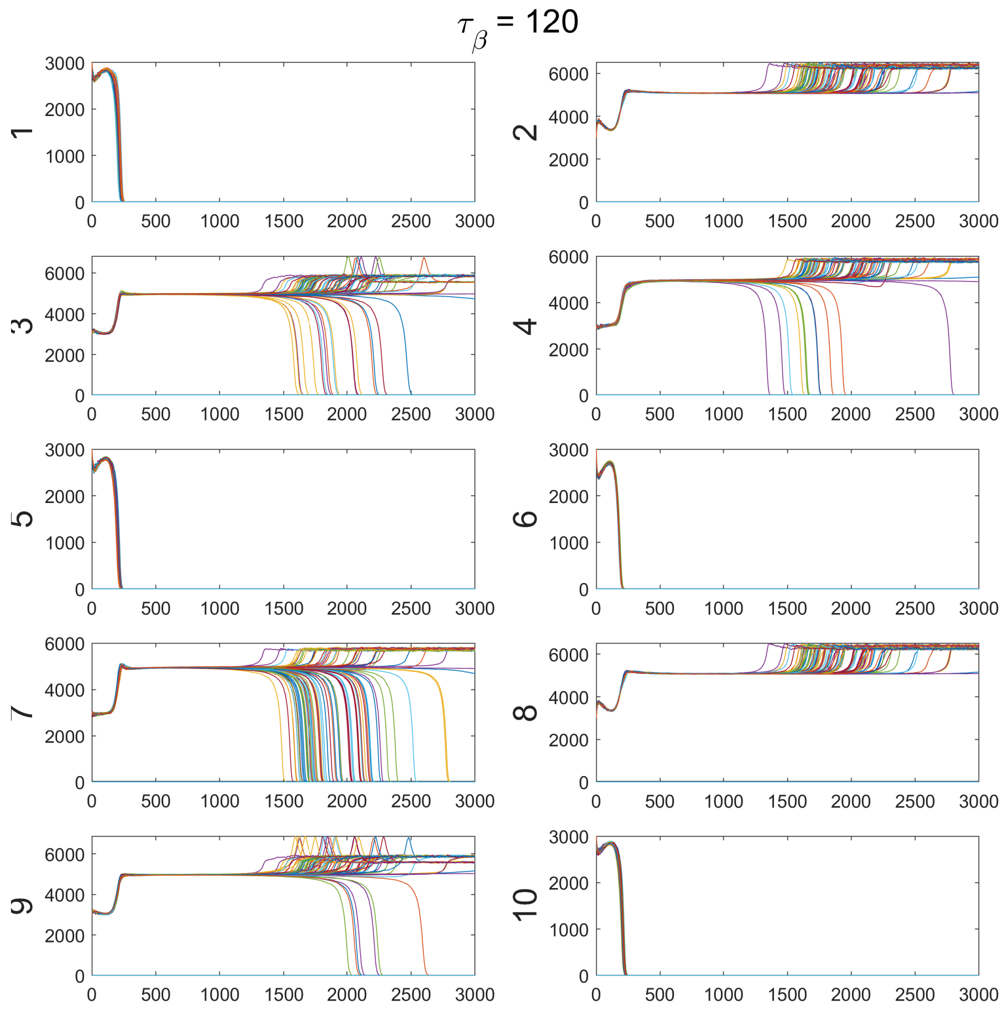

Fig S1.3. 100 realizations of the population time series of each city (1 to 10) for $\tau_\beta = 120, N = 3000,\ K_j = 5000,\ \delta = 0.2,\ \gamma = 50,\ \alpha = 1$, the same parameter set used in Figs. 2(c), 2(f), and 2(g).
